# Breathe easy, baby, breathe. Lung ultrasound in neonatal critical care

**DOI:** 10.3389/fped.2025.1631563

**Published:** 2025-09-23

**Authors:** Mateusz Jagła, Andrzej Grudzień, Tomasz Tomasik, Michał Wroński, Przemko Kwinta

**Affiliations:** Neonatal Intensive Care Unit, Pediatric Department, Jagiellonian University, Collegium Medicum, Krakow, Poland

**Keywords:** point-of care ultrasound, lung ultrasound, acute respiratory failure, neonate, neonatal transport

## Abstract

Lung ultrasound (LUS) has emerged as an essential tool in neonatology over the past two decades, offering unique advantages for this patient population. The small size, high water content, and delayed rib calcification of neonates make them particularly suited for ultrasonographic imaging. By replacing traditional chest radiographs, it significantly reduces exposure to ionizing radiation. Furthermore, it is widely accessible, easy to use, and provides repeatable, real-time imaging without requiring patient transport. These features make it invaluable in managing acute respiratory conditions, where timely intervention is critical. This review emphasizes the role of LUS in neonates with acute respiratory distress as a fundamental component of the point-of-care ultrasound (PoCUS) protocol. The technique is crucial for conditions such as respiratory distress syndrome (RDS), supporting decisions on surfactant therapy. It also aids in diagnosing and managing air-leak syndromes like pneumothorax (PTX) and detecting congenital respiratory malformations. Additionally, LUS ensures safer transport of critically ill neonates and optimizes mechanical ventilation. By delivering accurate, real-time imaging, LUS has become an essential diagnostic tool in infant care. Its integration into clinical practice enhances the management of life-threatening conditions, making it an essential skill for clinicians in neonatal intensive care units (NICU) and during neonatal transport.

## 1 Introduction

Lung ultrasonography has been a well-established imaging modality in clinical practice for many years. This review outlines the diagnostic and therapeutic applications of LUS in the management of critically ill neonates, based on the authors' clinical experience and evidence reported in scientific literature. Relevant literature was identified through Pubmed database searches using keywords such as *lung ultrasound, neonate, RDS, pneumothorax, congenital lung malformations, neonatal transport, and ultrasound-guided ventilation monitoring, crashing neonate*.

Lung ultrasonography, as a point-of-care ultrasound (PoCUS) modality performed at the bedside in intensive care units, was first introduced by Professor Daniel Lichtenstein through the “BLUE protocol” (1). In the follow-up to his original study, he confirmed the validity of fully transferring the method to the diagnosis of acute respiratory failure in newborns, stating that in terms of ultrasound examination *neonate lungs are a miniature version of an adult's lungs* (2).

Computed tomography (CT) remains the gold standard for detailed lung assessment in adults. However, its use in neonates, particularly those in critical condition—is limited due to concerns related to patient transport, the need for sedation, and exposure to ionizing radiation. LUS has become a standard tool in neonatal care, gradually replacing chest x-rays (CXR) in the evaluation of acute respiratory failure.

At the author's institution, LUS has been employed in routine diagnostics for approximately 15 years. Its safety and efficacy have been confirmed both in term and premature neonates, within NICU as well as during interhospital transport (3, 4). At present, LUS is routinely performed in our institution, with approximately 150–200 examinations conducted each month (our center is a Level III+ center with 30 intensive care beds. We provide care for a wide range of patients: extremely preterm newborns, newborns requiring hypothermia, surgery, neurosurgery, and cardiac surgery). In contrast, the use of CXR has been markedly reduced and is now limited to only a few cases per month, primarily for cardiological or surgical indications. The available literature demonstrates a clear advantage of LUS over chest CXR in the diagnostic accuracy of common causes of acute respiratory failure in neonates, including pneumothorax, pneumonia, pleural effusion, and pulmonary edema (1, 2, 5).

Lung ultrasound is simple to use and relatively easy to interpret, especially compared to other imaging methods. These features support its use in routine neonatal practice, even when operators have varying levels of experience (6). In addition, LUS allows for continuous monitoring of treatment effects and lung condition without repeated exposure to ionizing radiation.

This review examines the use of LUS as part of POCUS in neonates during their first days of life, covering ultrasound findings in acute neonatal respiratory conditions and congenital pulmonary malformations, as well as describing specific applications such as ventilation management, assessment of critically ill neonates, and neonatal transport.

## 2 Typical ultrasound findings in acute neonatal respiratory pathologies

### 2.1 Respiratory distress syndrome

**Figure 1:**
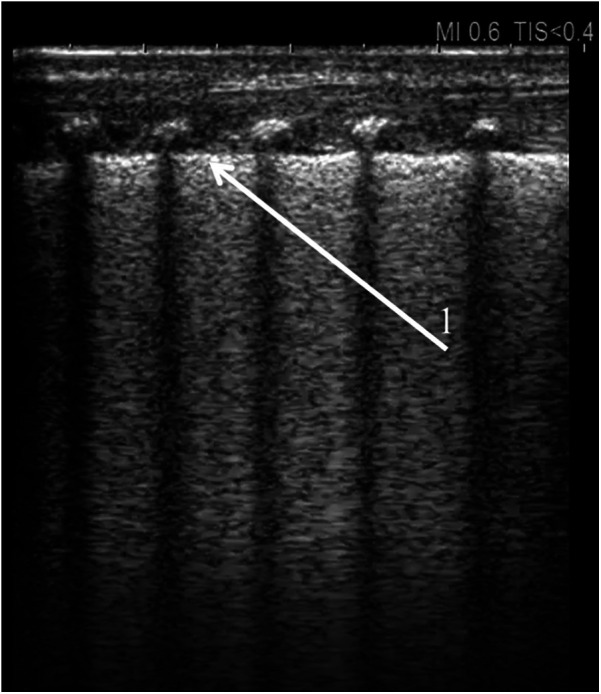
Universal alveolar-interstitial pattern (“white lung”). The female infant was delivered by Caesarean section at 27 weeks of gestation, with a birth weight of 1,150 grams. Her Apgar scores were 1, 2, 4, and 5, and she did not receive prenatal corticosteroids. Mechanical ventilation was initiated after delivery. First day of life. (1) Rough appearance of the pleural line. Linear probe, longitudinal scan.

**Figure 2:**
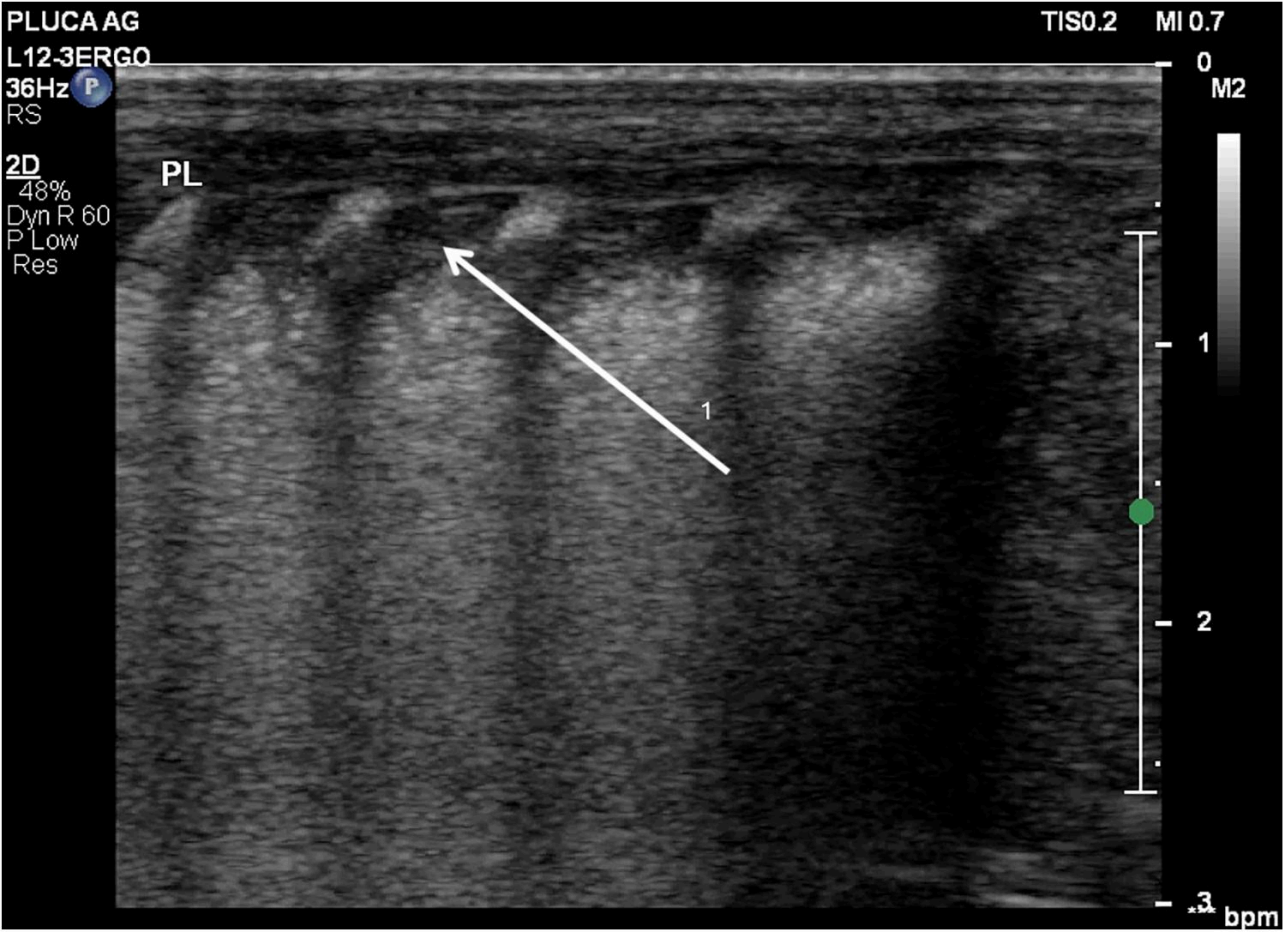
Diffuse alveolar-interstitial pattern (“white lung”) with subpleural thin consolidations. (1) Occurs in severe RDS. Linear probe, longitudinal scan. Same patient as Figure 1.

Surfactant deficiency syndrome in premature infants is a good example of the use of ultrasound as a diagnostic tool. In 2008, the Italian authors of one of the first publications demonstrated high sensitivity and specificity the LUS in identifying RDS. The atelectatic nature of the lung tissue, with varying degrees of interstitial thickening, is reflected on ultrasound in an increasing number of B-lines up to the image of an alveolar-interstitial pattern with subpleural consolidation (alveolar oedema, fluid alevologram) and deeper atelectasis in the most severe cases. Lung ultrasound seminology was published by Raimondi et al. (7). Identification of RDS with a sensitivity and specificity of 100% can occur if three abnormalities are present at the same time: alveolar-interstitial pattern (image of the “white lung”), pleural line abnormalities (small subpleural consolidations, thickening, irregularity and rough appearance) and affecting all areas of the lungs (Figures 1, 2. Supplementary Data—Videos S1, S2) (8).

The place of ultrasonography in diagnosing RDS and determining treatment has been established in the “European Consensus Guidelines on the Management of Respiratory Distress Syndrome: 2022 Update” in three areas: diagnosis of RDS, assessment of its severity, monitoring of complications and effectiveness of treatment. Currently, the indications for surfactant administration are based on clinical signs indicating respiratory distress in the newborn. Early use of nCPAP avoids the risks associated with endotracheal administration of surfactant. Deterioration of respiratory capacity will be an indication for surfactant administration (9).

In 2015, the RDS severity score scale, described by Brat et al., was used for the first time in the ultrasound evaluation of RDS (10). Over the years, there have been published studies using various modifications of this scale (both in terms of the number of lung fields analysed and the degrees of severity of the lesions). The value of various modifications of this scale is discussed in (11). The important limitation is the subjectivity in image interpretation, which may affect interobserver agreement, particularly in borderline or complex cases.

The ultrasound-based RDS severity scale (LUSs) is a tool that enables prediction of the need for surfactant therapy, repeat administration, escalation of respiratory support to mechanical ventilation, and even the risk of developing bronchopulmonary dysplasia (BPD) (10, 12–14).

The effects of administered surfactant can be monitored ultrasonographically (13). During endotracheal administration of the drug, displacement of the contents in the right and left bronchi can be observed, making sure of the symmetry of deposition in the lungs. Sometime after administration, it is advisable to assess the improvement of lung airflow—the appearance of A-line artifacts, reducing the number of B-lines. Due to the tendency to administer more drug to the right bronchus—it is possible to observe asymmetry of lung aeration—weaker aeration of the left lung. This heterogeneous picture indicates an increased risk of complications in the form of pneumothorax.

Asymmetric surfactant administration can result from deep position of the endotracheal tube. This can be prevented by assessing the position of the tube tip with ultrasound. A recent meta-analysis shows that LUS is a quick and effective technique for identifying the correct endotracheal tube position in neonates (15).

The study of lung recruitment maneuver in intubated patients with RDS under ultrasound guidance (ultrasound scale was used) was interesting. Reduction in the risk of atelectasis was associated with a better prognosis—faster reduction in oxygen requirements, shorter NICU stay, reduced inflammation, and shorter mechanical ventilation time (16).

### 2.2 Transient tachypnea of the newborn

Transient tachypnea of the newborn (TTN) is a frequent cause of perinatal dyspnea. TTN is characterized as a self-limited respiratory disorder resulting from delayed clearance of fetal lung fluid (17). The incidence of TTN ranges from 4% to 5.7% in full-term neonates and reaches approximately 10% in preterm infants (18). Although TTN is typically a temporary condition, the associated dyspnea can be clinically significant. The primary clinical challenge involves distinguishing TTN from other etiologies of neonatal respiratory distress, such as respiratory distress syndrome (RDS), pneumonia, and meconium aspiration syndrome. Copetti et al. introduced LUS for the diagnosis of TTN and defined a “double lung point” (DLP), which refers to the distinct boundary between lower lung regions displaying a hyperechoic, thin pleural line with compact B-lines and upper lung areas that are normal or nearly normal (19). Besides DLP, ultrasound findings of TTN include interstitial syndrome or “white lungs,” pleural line abnormalities, loss of A-lines, and pleural effusions (20). According to meta-analyses published in 2021, LUS demonstrated a pooled sensitivity of 0.67 (95% CI = 0.63–0.71) and specificity of 0.97 (95% CI = 0.95–0.98) for diagnosing TTN (21). A recent systematic review and meta-analysis conducted by Ma et al. reported that lung ultrasound (LUS) demonstrated a pooled sensitivity of 0.98 (95% CI: 0.92–1.00) and specificity of 0.99 (95% CI: 0.91–1.00), with an area under the curve of 1.00 (95% CI: 0.98–1.00) (22). Wang Y noted that DLP's effectiveness in diagnosing TTN differs widely across studies (sensitivity 100–45.6%, specificity 100–94.8%). Using DLP together with B-line as a diagnostic standard may greatly enhance LUS accuracy (23).

### 2.3 Air leak syndromes (ALS)

Considering recent reports, LUS demonstrates up to 100% sensitivity and specificity in the diagnosis of PTX in neonates (24–27). It has been included in the recommendations of the PoCUS Working Group of the European Society of Paediatric and Neonatal Intensive Care (ESPNIC) regarding the diagnosis and treatment of pneumothorax (28). Lung PoCUS, including the exclusion of PTX, is also an integral part of protocols such as the Crushing Neonate Protocol (CNP) and the Sonographic Assessment of liFe-threatening Emergencies—Revised (SAFE-R) (29, 30). Among the key advantages of ultrasound in AIS—particularly PTX—are its ready availability and rapid execution, significantly shortening the time needed for diagnosis (31, 32).

**Figure 3:**
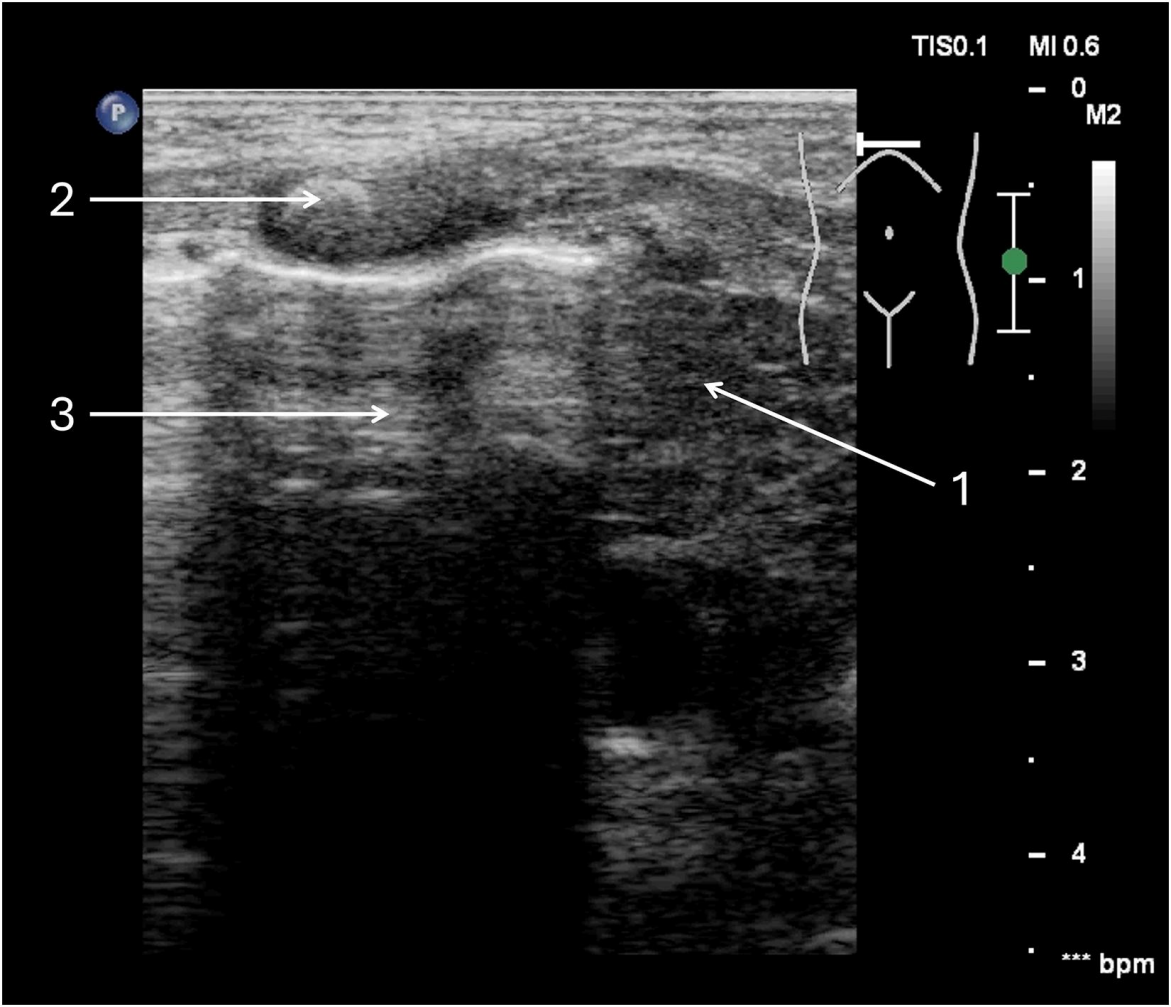
Tension pneumothorax of the right pleural cavity. A full-term neonate, two days of age, diagnosed with congenital pneumonia, was initiated on nasal continuous positive airway pressure (nCPAP) two hours prior. (1) Thymus—mediastinum displaced to the left side. (2) Ossification nucleus in the sternum. (3) free air chamber in the pleural cavity on the right side.

For ultrasound in suspected PTX, a high-frequency linear probe is used, and the neonate is positioned supine. The first step in the evaluation is assessing the position of the mediastinum (especially the thymus, which is easily recognizable on ultrasound) and ruling out any significant shift of mediastinal structures relative to the centrally located sternum, which is characterized by a hyperechoic ossification nucleus and a posterior acoustic shadow (Supplementary Data—Video S3). A significant shift of mediastinal structures toward the midline, caused by the free air chamber, allows for the diagnosis of tension pneumothorax (Figure 3). The anterior transverse plane showing loss of mediastinal structures and visible A-lines beneath the sternum demonstrates high diagnostic accuracy: sensitivity 99.0%, specificity 100%, PPV 100%, and NPV 98.8% (33). When the transducer is placed over the lung fields in the case of pneumothorax, the following findings are expected: absence of the normal pleural sliding sign, an overrepresentation of A-line artifacts (reverberations at the air pocket interface), absence of B-line artifacts (which arise due to small amounts of fluid in interlobular septa), and absence of lung pulse—the transmission of cardiac pulsations to the pleural line (25, 26).

**Figure 4:**
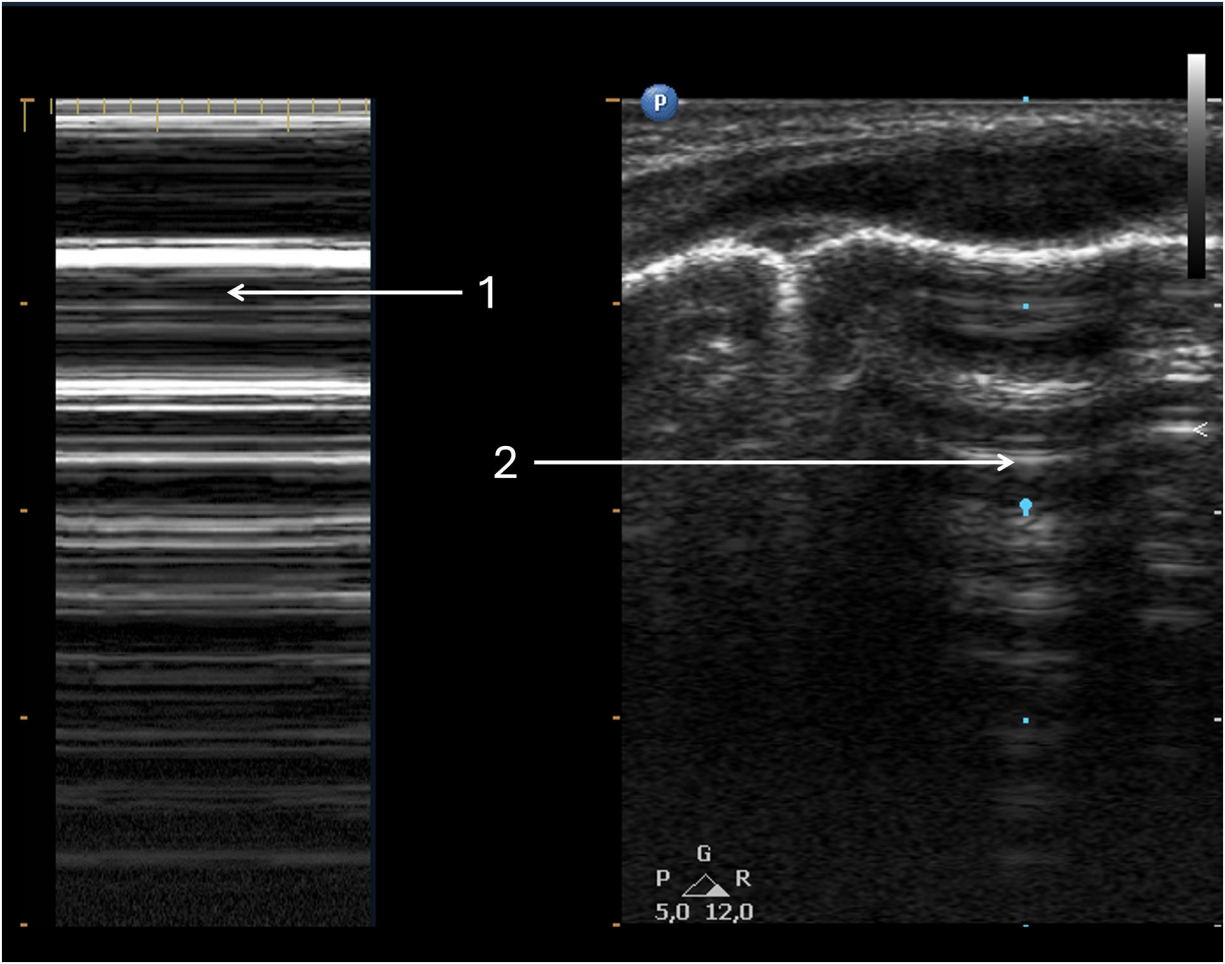
Pneumothorax. Same patient as Figure 3. (1) Pneumothorax in M-mode option—“barcode” or “stratosphere” image. (2) Pneumothorax in 2D presentation.

**Figure 5:**
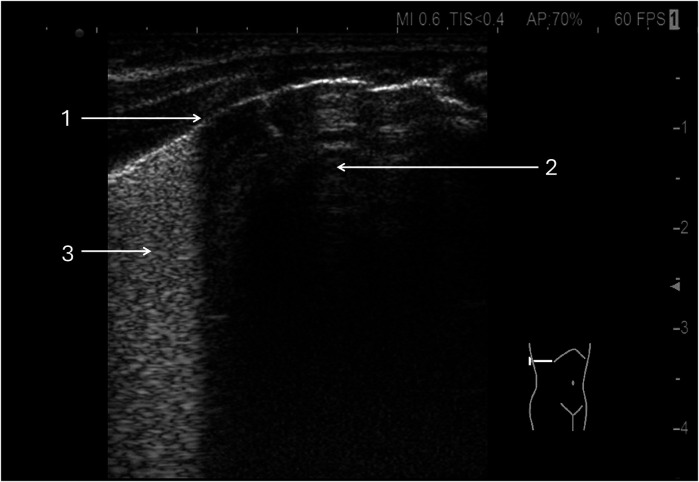
Lung point in pneumothorax. A 32-week premature infant was resuscitated at birth with an Ambu bag. (1) Lung point. (2) Free air chamber in the right pleural cavity. (3) Confluent B lines in the aerated lung.

In uncertain cases, diagnostic accuracy can be enhanced using M-mode ultrasound: in PTX, the overrepresented A-lines below the pleural line will create the so-called “barcode sign” or “stratosphere sign” (Figure 4), replacing the normal granular pattern known as the “seashore sign”. A key advantage of lung ultrasound is the ability to precisely locate the border of the air pocket by identifying the so-called lung point—the site where pleural sliding disappears (Figure 5, Supplementary Data—Video S4) (26, 27). If decompression of the pneumothorax is necessary, the exact margins of the trapped air in the pleural cavity can be accurately determined after properly positioning the infant, thereby guiding the site for needle insertion or chest tube placement (34).

Ultrasound can also be effectively used to verify the outcome of pneumothorax decompression. Indicators confirming successful drainage or aspiration include the return of mediastinal structures to the midline, disappearance of the lung point, reappearance of pleural sliding, and the presence of B-line artifacts. Additionally, the presence of the chest tube in the pleural cavity can be confirmed by visualizing its passage through the parietal pleura between the ribs.

The presence of free air in the mediastinum is most often an incidental finding accompanying signs of respiratory distress during PTX or pulmonary interstitial emphysema (PIE). In ultrasound, pneumomediastinum (PM) should be suspected when free air in the mediastinum (overrepresented A-line artifacts due to reverberations at the air interface) prevents evaluation of mediastinal structures, particularly the heart, during echocardiographic examination (Supplementary Data—Video S5). In most cases, this is a transient phenomenon that does not require any invasive intervention. Simultaneous visualization of the medial lung borders, with the presence of the pleural sliding sign and B-line artifacts, excludes the presence of PTX and allows differentiation between PM and PTX.

### 2.4 Consolidation

Consolidation refers to lung tissue changes from fluid-filled or collapsed alveoli. It appears on ultrasound when just beneath the pleural line and can result from inflammation, bleeding, obstruction, or external pressure. Atelectasis is consolidation due to pressure. Small subpleural consolidations are seen in severe RDS. Bronchograms, whether static or dynamic, may be seen in larger consolidations (35). In cases of meconium aspiration syndrome, consolidations may be irregularly distributed throughout the lungs, appearing adjacent to normal tissue or as an alveolar-interstitial pattern. Consolidation can present with bronchograms or show evidence of atelectasis resulting from a meconium plug (Supplementary Data—Video S6: Transversal linear probe ultrasound shows diffuse lung consolidations due to meconium aspiration syndrome. The patient is a term female (40 weeks, delivered by caesarean section) with birth weight 3,160 g and Apgar scores of 1, 4, 5, and 6.).

Acute Respiratory Distress Syndrome (ARDS) is characterised by a diffuse alveolar-interstitial pattern on imaging, accompanied by areas of consolidation with air bronchograms or evidence of atelectasis (7). Lung ultrasound findings for pneumonia include large consolidations with irregular margins and air bronchograms, pleural line abnormalities, interstitial syndrome, and pleural effusion (36). In neonates, lung ultrasound showed 96% sensitivity and 100% specificity for diagnosing pneumonia, though this is based on just two studies and may reflect selection bias (37). Newer research indicates that while lung ultrasound effectively assesses the presence and severity of neonatal pneumonia by measuring consolidation extent, it cannot identify aetiology (pathogens) in early disease stages (38). Inflammatory consolidation remains unchanged with posture or recruitment maneuvers and is associated with a bronchogram. In summary, analysis of lung consolidations in newborn ultrasound should consider the clinical context.

### 2.5 Pleural effusion

Lung ultrasound is the main method for diagnosing pleural effusion in newborns. Though uncommon (affecting less than 2.2% of NICU cases), pleural effusion can be life-threatening and has various causes. Most prenatal cases are linked to congenital chylothorax, severe heart disease with hydrops fetalis, or serious intrauterine infections. In the postnatal period, life-threatening effusions most commonly occur as a result of iatrogenic causes, such as thoracic surgical procedures or complications related to central venous catheterization, or may arise secondary to severe respiratory infections (39). The assessment for pleural fluid is an established element of point-of-care ultrasound (PoCUS) protocols during the evaluation of acute clinical deterioration in neonates within the NICU, as described in The Crashing Neonate section.

**Figure 6:**
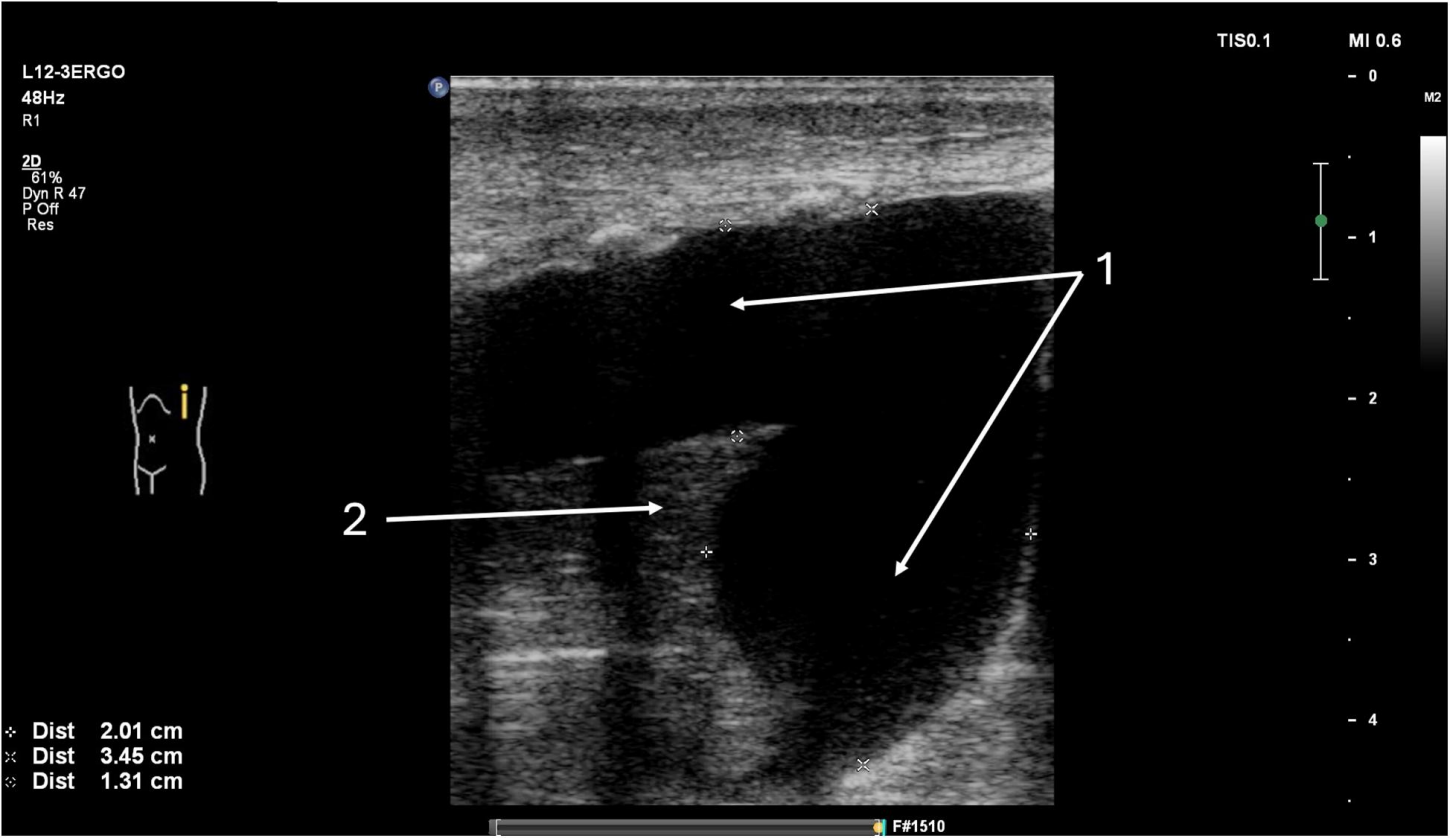
Fluid detected in the left pleural cavity, longitudinal lung ultrasound. The patient is a 10-day-old newborn with coarctation of the aorta following cardiac surgery involving repair of the aortic arch. (1) Large volume of fluid present in the left pleural cavity. (2) Complete atelectasis of the lower lobe of the left lung.

On ultrasound, pleural fluid is seen as a hypoechoic area between the parietal and visceral pleura. It may be free or loculated, localized or diffuse, and can appear homogeneous or with fine hyperechoic particles (“plankton sign”) (40). Quantification usually involves measuring the gap between the lung base and diaphragm or between the lateral lung surface and chest wall (see Figure 6). Large unilateral effusions may shift the mediastinum to the opposite side. The compressed lung often displays prominent B-lines or appears consolidated. Extensive effusions can clinically resemble pneumothorax.

## 3 Congenital pulmonary malformations

Lung ultrasound is often the first-line imaging modality in neonates with respiratory distress. The initial step of the examination involves assessing the position of mediastinal structures. Mediastinal shift is most commonly associated with tension PTX but may also result from less common causes, such as congenital diaphragmatic hernia or rare congenital pulmonary malformations. LUS has shown high concordance with chest CT in the evaluation of congenital lung abnormalities, as demonstrated by Yousef et al. (41). Point-of-care ultrasound is crucial when prenatal diagnostic data are lacking, and chest CT is not available prior to transfer to a tertiary care center. In such cases, proficiency in ultrasound is essential to avoid misdiagnosing structural anomalies as pneumothorax and to prevent inappropriate chest drain placement. LUS is increasingly recognized as an essential modality not only for the diagnosis of congenital respiratory malformation in neonates, but also for longitudinal monitoring of lesion evolution and the identification of coexisting structural anomalies (42). The ultrasonographic appearance of pulmonary lesions reflects their anatomical and pathological characteristics, including location, vascularization, impact on surrounding lung tissue, and underlying mechanism of formation.

### 3.1 Congenital diaphragmatic hernia

**Figure 7:**
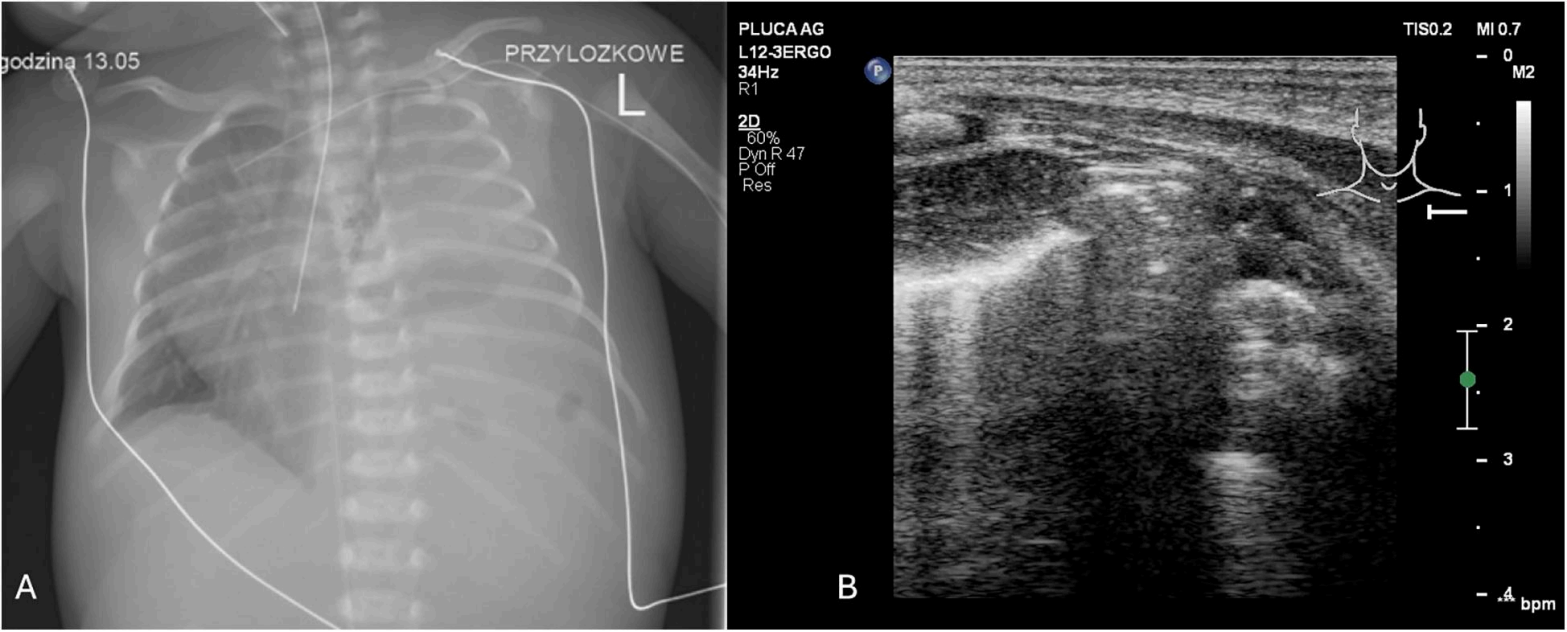
**(A)** CXR. Left-sided congenital diaphragmatic hernia. **(B)** LUS. Left side of sternum lacks pleural line, polycyclic echoes corresponding to intestinal echostructure (1). Transversal position of the linear probe. A female infant was delivered via C-section at 39 weeks, weighing 3,000 g with Apgar scores of 4/6/6/7. She required immediate intubation, SIMV with 100% O2, surfactant therapy, and presented with severe pulmonary hypertension, systemic hypotension, and heart failure.

Congenital diaphragmatic hernia (CDH) is the displacement of abdominal organs into the chest by a defect of the diaphragm. On the right side, the hernia typically contains a portion of the liver, which must be carefully differentiated from atelectatic lung tissue. The contents of the left-sided hernia are usually the stomach, intestines, and spleen. Intestines initially airless, quickly fill with air swallowed by the newborn—pleural line disruption, polycystic contours and peristaltic movements can be observed. Through the acoustic windows of the airless intestines, it is possible to visualize the vessels running in the mesentery drawn into the thorax. Additionally, by following the echotexture of the diaphragm, the margin of the defect can be delineated (Figure 7, Supplementary Data—Videos S7, S8).

Large defects result in hypoplasia of the compressed or both lungs and pulmonary hypertension of an irreversible nature. Small hernias in naturally occurring orifices (posterior-lateral—Bochdale's—more common, including more common on the left side, and anterior—Morgagni's) proceed without clinical symptoms (43).

Italian researchers modified the LUS scale to assess newborns with CDH, enabling physicians to tailor ventilation therapy around surgery. The adapted scale evaluates six areas per lung, providing objective data to determine optimal surgery timing and guide weaning from mechanical ventilation (44).

Postoperative lung assessment is commonly performed using lung ultrasound (LUS), which enables evaluation of lung expansion and detection of complications such as pneumothorax, pleural effusion, and consolidations indicative of pneumonia. The duration of abnormal LUS patterns may have prognostic value. For patients extubated within 7 days, the pattern typically returns to normal within 48 h following surgery. In contrast, patients who require prolonged ventilation often exhibit an interstitial or alveolar-interstitial pattern in both lungs for 2–3 weeks.

LUS may reveal features such as atelectatic cystic structures, irregular fluid-filled cysts, solid heterogeneous consolidations, emphysematous cysts, or abnormal vascular patterns—findings that can suggest the presence of CDH or congenital pulmonary malformation.

### 3.2 Congenital pulmonary airway malformation

Congenital pulmonary airway malformation (CPAM), formerly known as congenital cystic adenomatoid malformation (CCAM), is the most common type of congenital lung malformation, accounting for approximately 30%–40% of cases (45). Usually unilateral, affects part of the lung, a lobe, or the entire lung. Non-respiratory lung tissue with undeveloped alveoli arises from uncontrolled hypertrophy of the terminal bronchioles. CPAM is usually solid in nature, with small air spaces or a cystic appearance. The cysts communicate with the surrounding parenchyma. Due to the concept of the formation of cystic lesions at different levels of the airways, there are now various histopathological types of CPAM (46–48). Type I is the most common form and is characterized by one or more large cysts (>2 cm in diameter) with a thick muscular wall. Type II lesions consist of uniform cysts measuring 0.5–1 cm in diameter, with thin walls. They are often associated with other congenital anomalies, including cardiac, renal, and diaphragmatic defects, and are linked to a poor prognosis. Type III represents a generalized adenomatoid overgrowth involving an entire lobe or multiple lobes. It appears as a solid mass containing small cysts (0.3–0.5 cm) and is considered the most severe form of CPAM due to its extensive nature, which can cause compression of adjacent structures, mediastinal shift, and even prenatal hydrops from cardiovascular compromise (46). Type IV consists of peripheral cystic lesions that histologically resemble type I well-differentiated pleuropulmonary blastoma (47).

Some forms of CPAM have communication with the bronchial tree, while others are associated with bronchial stenosis or atresia. Prenatal ultrasound diagnosis is possible. Rapid prenatal (at 20–26 weeks' gestation) development of the lesion may lead to impaired venous outflow, heart failure and generalized fetal hydrops. Fetal surgery is a potential therapeutic option in selected cases.

**Figure 8:**
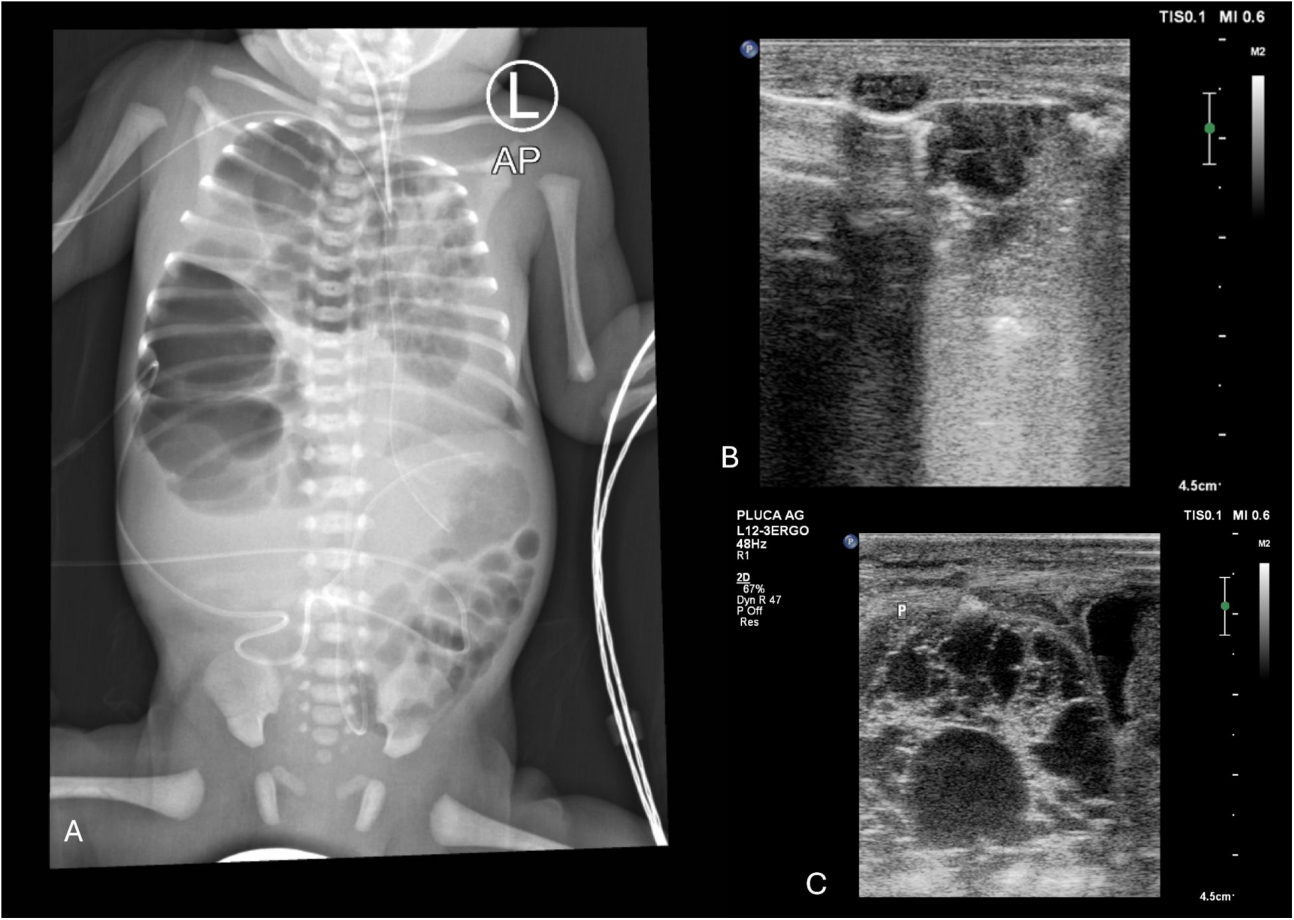
**(A)** CXR. Right-sided CPAM, type III on histological examination. Heterogeneous right lung distension causing mediastinal shift to the left side (1. endotracheal tube position), with signs of pressure on the left lung. **(B)** LUS. Right lung distention—a fluid cystic lesion in the medial upper part of the right lung displaced to the left side relative to the sternum. Emphysematous bulla (2). Fluid cystic lesion (3). Longitudinal position of the linear probe. **(C)** LUS. Fluid cystic lesion in the lower part of the right lung. Longitudinal position of the linear probe. A male infant, delivered by caesarean section at 31 weeks (birth weight: 1950 g) with prenatally diagnosed CPAM and polyhydramnios, required intubation, repeated surfactant administration, and mechanical ventilation postnatally. Despite high-frequency oscillatory ventilation (FiO_2_ 1.0), nitric oxide, vasopressors, and epoprostenol for PPHN and hypotension, the left lung remained abnormal after surgery without expansion. The patient died.

In postnatal lung ultrasound CPAM appears as cysts, emphysematous bullae, and glandular changes of a non-homogeneous consolidation nature. The vascularization of these changes most often comes from the pulmonary artery, venous drainage to the pulmonary venous system (Figure 8).

### 3.3 Bronchopulmonary sequestration

Bronchopulmonary sequestration (BPS), accounting for approximately 6% of congenital lung malformations, must be carefully differentiated from other atelectatic lesions (42). The sequestered segment is typically supplied by an aberrant artery arising from the thoracic or abdominal aorta, lacks communication with the airway, and does not participate in gas exchange. Depending on the type and extent of the lesion, systemic blood flow to the area may be increased.

**Figure 9:**
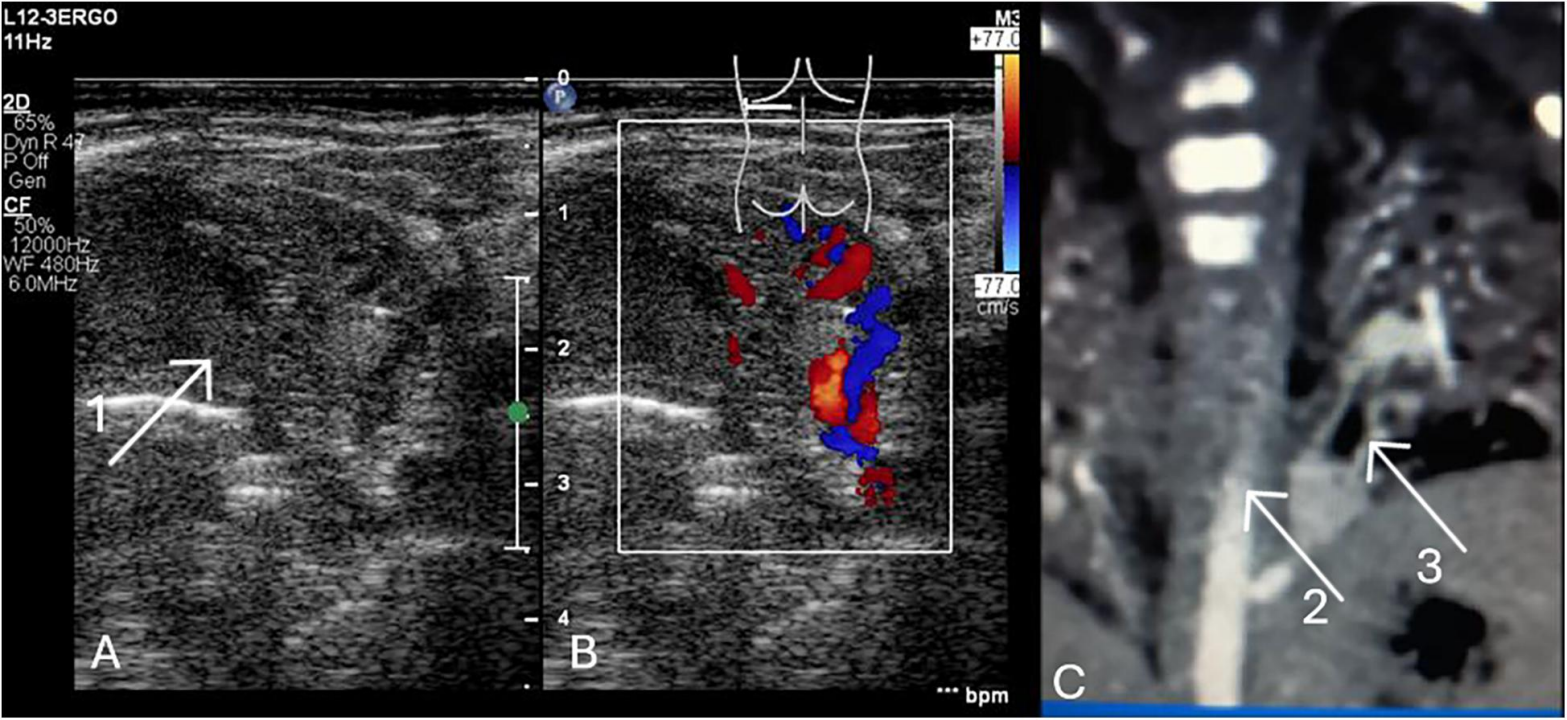
**(A)** LUS. Intrapulmonary sequestration—atelectatic area with heterogeneous structure, mostly solid (1). Transversal position of the linear probe, supradiaphragmatic region of the posterior field of the left lung. **(B)** LUS. The vessels marked with color Doppler are branches of the aorta. **(C)** Angio CT. The lesion (2). Aortic branch (3). The male neonate was admitted with respiratory failure (nCPAP, FIO_2_ 0.4, RDS) following caesarean section at 37 weeks gestation, with a birth weight of 3,320 g and an Apgar score of 9. The mother received lamotrigine and lacosamide for epilepsy during pregnancy. Prenatal imaging indicated possible pulmonary sequestration. Echocardiography revealed an atrial septal defect and a right subclavian artery arising from the aortic arch. The respiratory disorder resolved, and the patient was discharged with a plan for outpatient follow-up at a surgical clinic in 2–3 months to assess and schedule resection of the pulmonary sequestration.

Suspicion of BPS should be made prenatally. Ultrasound performed after birth can facilitate the identification of atelectatic lesions, which are most commonly located in the posterior basal segments, just above the diaphragm. Bronchopulmonary sequestration may present as intralobar or as extralobar, depending on its anatomical separation and pleural covering (Figure 9, Supplementary Data—Video S9).

Intrapulmonary sequestration (ILS) is located in the visceral pleura, most often supradiaphragmatic in the posterior field of the left lung. Ultrasound can detect a solid, cystic, cavernous, or cirrhotic lesion. The pleura is fibrotic. Arterial vascularization through atypical branches from the thoracic or abdominal aorta. Venous drainage into the pulmonary venous system. This type of defect is not associated with other defects.

Extrapulmonary sequestration (ELS) is surrounded by its own pleura, completely separated from healthy lung tissue. It is located supradiaphragmatically, in the diaphragm muscle, or subdiaphragmatically—in the retroperitoneal space in the area of the left adrenal gland. It requires differentiation from neuroblastoma (49). Arterial vascularization from the aorta, venous outflow to systemic veins or to the left atrium. This defect can be associated with other heart defects or pulmonary hypoplasia (47). In the case of changes involving the vascularization, it is most often necessary to perform angio CT of the chest. Sometimes congenital malformations may be of a hybrid nature, combining features of sequestration and CPAM. Foregut-derived bronchopulmonary malformations involve a sequestered segment of lung tissue that communicates with the gastrointestinal tract, typically the distal oesophagus or stomach. These lesions are more commonly located on the right side and are frequently associated with CDH, the VACTERL association, and clinical manifestations such as recurrent pneumonia, chronic cough, and respiratory distress (47).

### 3.4 Congenital lobar emphysema

Congenital lobar emphysema (CLE) accounts for 10% of congenital lung defects. It is a distention of a lobe or part of a lobe, most often the left upper lobe, caused by congenital bronchial stenosis. Symptoms appear after birth—increasing emphysema due to air trapping. If the lesion affects the entire lobe—it enlarges to such an extent that it causes displacement of the mediastinal structures to the opposite side, with impaired aeration of the compressed normal lung tissue with the risk of tension pneumothorax. LUS examination reveals mediastinum displacement, pulmonary hyperinflation (predominance of A-line)—displacement of the edges, decreased mobility of the lower edge, disappearance of the sliding sign or pneumothorax.

Atelectatic changes (BPS) require differentiation between inflammatory consolidations, neoplastic or vascular changes. Expansive lesions with mass effect (CPAM, CLE, CDH) cause displacement of the mediastinum to the opposite side, which is easily detected using ultrasound. These patients will most likely require surgical intervention (45).

## 4 Role of LUS in neonatal respiratory management

Neonate suffering from acute respiratory failure may require noninvasive breathing support or assisted ventilation. The following section outlines how LUS can support the optimization of ventilation in NICU.

### 4.1 Noninvasive ventilation (NIV)

LUS can help determine the indications for NIV and monitor its effectiveness. The underlying principle of lung imaging using ultrasound is that the more B-lines are present in the lung image, the worse the lung aeration. Based on this information, in a newborn with desaturation, it will be easier to select the group that will require respiratory support with positive end-expiratory pressure (PEEP). In subsequent steps, ultrasound will help with assessment of the effectiveness of NIV by differentiating patients with improved lung imaging (reduction in B-line profile) from those who benefit from escalation of respiratory support.

Since 2022, according to European guidelines for the care of preterm newborns, the LUS score is a factor that helps determine which preterm infants, currently on noninvasive respiratory support, should receive intratracheal surfactant (9, 13, 50, 51). Numoerous studies suggest LUS score can be used to predict the necessity of escalation to mechanical ventilation in preterm infants requiring respiratory support (51, 52–56).

### 4.2 Mechanical ventilation (MV)

During the management of a patient on MV, constant and accurate monitoring of their general condition, pulmonary status, and ventilation effectiveness is necessary. LUS is increasingly used as a complementary method alongside standard monitoring tools such as physical examination, blood gas analysis, and the oxygenation index. LUS performed in newborns receiving assisted ventilation allows for the assessment of lung aeration, including the identification of overdistension or areas of atelectasis. LUS is also known as an effective tool in assessment of lung recruitment maneuvers, which are performed as a rescue approach in critically ill neonates (16, 56–58). As a bedside, real-time, and safely repeatable examination, it enables monitoring of the effects of therapeutic interventions, such as changes in ventilator settings, endotracheal tube and patient positioning, or administration of mucolytic agents.

Another use of LUS has been reported that in patients with a particularly severe manifestation of respiratory failure in the course of acute respiratory distress syndrome (ARDS). Ultrasound enables the choice of appropriate ventilation pressures (PEEP), resulting in a reduction in the frequency of complications and shortening the duration of mechanical ventilation, better or as well as previously used clinical tools (59, 60).

Algorithms for extubation also incorporate lung ultrasound. In a hemodynamically stable patient, after sedation withdrawal, a pressure support breathing trial is performed through the endotracheal tube, and by assessing the degree of lung aeration in the following hours, the success of weaning from mechanical ventilation can be predicted (61). LUS score also appears to be helpful in predicting extubation failure, though its accuracy may be lower in extremely preterm infants (62, 63) To date, no studies have explored the use of LUS in patients on high frequency ventilation. From the authors' experience, some of the rules, such as the evaluation of lung aeration or atelectasis presence, can be transferred from those that are used for conventional ventilation.

## 5 The crashing neonate

Point-of-care ultrasound guidelines offer standardized approaches for the suddenly decompensating infant in the NICU for the most common complications requiring urgent intervention. Several PoCUS protocols have been developed to address specific diagnostic and therapeutic challenges in neonatal intensive care, aiming to improve the safety and efficiency of critical care through rapid bedside assessment and early identification of life-threatening conditions (28–30).

One notable example is the Crashing Neonate Protocol (CNP), proposed by Elsayed et al., which was specifically designed for neonates with severe cardiorespiratory instability. Applicable to both term and preterm infants, the CNP complements existing neonatal resuscitation guidelines through a structured, stepwise ultrasound-based approach. A related model, the SAFE-R protocol developed by Yousef et al., focuses on rapidly assessing critically ill neonates who suddenly decompensate in the neonatal intensive care unit. Like CNP, it integrates focused cardiac, pulmonary, abdominal, and cranial ultrasound (29, 30).

In addition, international evidence-based guidelines issued by the PoCUS Working Group of the ESPNIC provide comprehensive recommendations for ultrasound evaluation of the heart, lungs, brain, abdomen, and vasculature (28).

Another protocol, modified algorithm for life-threatening emergencies, proposed by Ibarra-Ríos D et al, can be applied in the neonatal intensive care and the delivery room in relation to three scenarios: cardiac arrest, hemodynamic deterioration, or respiratory decompensation (64).

These guidelines have been validated for clinical use and represent a significant step toward the standardization of PoCUS in neonatal and pediatric critical care. However, many of the current recommendations are based on moderate or low-quality evidence or expert opinion, due to the limited availability of high-quality studies in this population. Broad clinical questions have been used to evaluate the value of PoCUS in areas such as cardiac, pulmonary, cerebral, abdominal, and vascular assessment, acknowledging that randomized controlled trials specifically measuring outcome improvements are scarce.

Certain PoCUS applications may not yet be feasible in all clinical settings, particularly those involving extremely preterm or non-sedated infants, which require advanced operator skills. Nevertheless, the existence of formal PoCUS guidelines is expected to promote local expertise development, stimulate future research, and help standardize clinical practice.

## 6 Neonatal transport

Point-of-care ultrasound has been utilized in pre-hospital and emergency settings, including transport of critically ill patients, since the 1990s. One of the earliest documented uses was the deployment of portable ultrasound devices during helicopter missions, demonstrating feasibility for diagnoses such as pneumothorax, hemothorax, pericardial effusion, or hypovolemia directly on-site. Most current guidelines on pre-hospital PoCUS focus on adult patients (65, 66).

PoCUS is increasingly used in highly specialized neonatal transport teams, particularly in the management of critically ill infants. For more than a decade, it has been applied in both ground and air interfacility medical transport settings (67–69). The use of point-of-care ultrasound in neonatal care during interfacility transport is gaining clinical relevance; nonetheless, the evidence base remains limited, and relevant publications and guidelines are scarce and rarely published (70).

Early reports supporting the feasibility of ultrasound application in neonatal transport began to emerge over a decade ago (69, 71). A major advantage of PoCUS in the transport setting lies in its capacity for rapid, bedside or incubator-side diagnostics, which facilitates immediate therapeutic decision-making. The example of the practical application of ultrasound during neonatal transport is its use in the rapid exclusion of pneumothorax, which was possible in over 90% of cases. This enabled timely decisions such as endotracheal intubation or ventilatory adjustment, contributing to more effective stabilization during transfer (4).

Ultrasound equipment used during transport must be compact, battery-operated, and user-friendly. Linear and sector transducers are the most commonly used. Advancements in technology allow for the use of relatively low-cost, wireless devices capable of rapid image transmission. This feature is particularly valuable in cases of diagnostic uncertainty, as it enables remote consultation with more experienced clinicians in tertiary centers (72).

Ultrasound is particularly helpful in identifying pulmonary causes of acute respiratory failure, such as RDS or PTX, and cardiovascular conditions including shock or critical congenital heart defects. It can also be used to monitor the effectiveness of interventions such as surfactant administration, ventilator adjustments, and endotracheal tube positioning (28).

Many centers have implemented structured training pathways for neonatal PoCUS operators, which include supervised practice, formal courses, and clinical application within NICUs (73). Our transport team routinely uses the simplified SAFE-R protocol, which includes focused evaluation of the lungs, heart, and great vessels in hemodynamically unstable neonates. This protocol allows for rapid identification of the most common causes of sudden clinical deterioration and real-time assessment of the response to treatment (30).

Despite the many advantages of PoCUS in critical neonatal transport, current evidence is limited by several factors: most data come from single-centre observational studies and lack of protocol validation. Additional limitations include the absence of blinded results interpretation and the potential bias from prior clinical knowledge of the patient's condition. Nevertheless, there is a clear need for well-designed, multicentre prospective studies to further confirm the safety, feasibility, and clinical impact of PoCUS in neonatal transport.

## 7 Summary

Lung ultrasound is a safe, bedside imaging modality increasingly used in the assessment of neonatal respiratory failure. It offers real-time evaluation without radiation exposure and can be performed repeatedly to guide clinical decisions. Its routine use improves diagnostic accuracy, facilitates respiratory management, and enhances the quality of care in neonatal intensive care units. This review summarizes current evidence from the literature and integrates the authors' extensive clinical experience with LUS in neonatal care. However, further evidence-based data are needed to validate standardized protocols and fully define the role of this novel diagnostic modality.

## Supplementary material

Supplementary Video S1 Examination using a 12Mhz linear probe, transversal orientation. The picture shows that the right lung is better aerated than the left one. It caused by the preferential administration of surfactant to the right bronchus. The video was obtained at the female infant, who was delivered by Caesarean section at 27 weeks of gestation, with a birth weight of 1150 grams. Her Apgar scores were 1, 2, 4, and 5, and she did not receive prenatal corticosteroids. Mechanical ventilation was initiated after delivery. The first day of life.

Supplementary Video S2 Examination using a 12Mhz linear probe, longitudinal orientation. The imagine shows the left lung with severe RDS (diffuse alveolar-interstitial pattern “white lung”) and next sequence—the right lung, which is better aerated than the left one. Compare the mobility of the lower border of the lungs, indicating better compliance of the right lung. The video was obtained at the female infant, who was delivered by Caesarean section at 27 weeks of gestation, with a birth weight of 1150 grams. Her Apgar scores were 1, 2, 4, and 5, and she did not receive prenatal corticosteroids. Mechanical ventilation was initiated after delivery. The first day of life.

Supplementary Video S3 Tension pneumothorax of the right pleural cavity, transversal lung ultrasound. A full-term neonate, two days of age, diagnosed with congenital pneumonia, was initiated on nasal continuous positive airway pressure (nCPAP) two hours prior. (Same patient as Figure 3). The imaging reveals a significant free air chamber within the right pleural cavity, characterized by prominent A-line artifacts and absence of the pleural sliding sign. This finding is associated with mediastinal shift towards the left side relative to the sternum. Additionally, a hyperechoic ossification nucleus is observed, as indicated by the structure labels in Figure 3.

Supplementary Video S4 Lung point in pneumothorax, transversal lung ultrasound. A 32-week premature infant was resuscitated at birth with an Ambu bag. (Same patient as Figure 5). The film displays part of the aerated lung contacting the pulmonary pleura, showing the pleural sliding sign on the left and a free air chamber with prominent A-line artifacts and no sliding sign on the right. The border where the sliding sign ends is known as the lung point (see Figure 5).

Supplementary Video S5 Pneumomediastinum: On transverse lung ultrasound, excessive A-line artifacts indicate mediastinal free air due to reverberation at the air interface (arrow). This free air obscures assessment of mediastinal structures, especially the heart.

Supplementary Video S6 The investigation shows diffuse lung consolidations due to meconium aspiration syndrome (MAS). The patient is a term female (delivered by caesarean section, at the GA of 40 weeks, with birth weight 3160 g and Apgar scores of 1, 4, 5, and 6). Short, rapid movements of the pleural line are associated with the high-frequency ventilation (HFO).

Supplementary Video S7 Left-sided congenital diaphragmatic hernia. Examination was performed using a 12 MHz linear probe in a transverse orientation. The image demonstrates mediastinal shift to the right, a portion of aerated left lung, peripheral absence of a normal pleural line, and intestinal loop echoes. The patient is a female infant delivered via cesarean section at 39 weeks gestation, with a birth weight of 3000 g and Apgar scores of 4/6/6/7. She was intubated immediately, placed on SIMV with 100% O2, received surfactant therapy, and exhibited severe pulmonary hypertension, systemic hypotension, and heart failure.

Supplementary Video S8 Left-sided congenital diaphragmatic hernia. The image reveals intestinal loops in the left chest, some gas-filled, others not. A female infant was delivered by C-section at 39 weeks (weight 3000 g; Apgar scores 4/6/6/7). She required immediate intubation with SIMV and 100% O₂, surfactant therapy, and developed severe pulmonary hypertension, systemic hypotension, and heart failure.

Supplementary Video S9 Left panel: CXR—non-diagnostic. Right panel: Angio CT. An atelectatic lesion was identified in the lower lobe of the left lung, with arterial supply originating from a branch of the aorta. Bottom panel: LUS. The atelectatic lesion in supradiaphragmatic part of the lower lobe of the left lung. On the right—colour Doppler to visualize the vessels supplying blood from the aorta to the lesion. A male neonate presented with respiratory failure (nCPAP, FIO2 0.4, RDS) following caesarean delivery at 37 weeks’ gestation; birth weight was 3320 g and Apgar score was 9. The mother had received lamotrigine and lacosamide for epilepsy throughout pregnancy. Prenatal imaging suggested possible pulmonary sequestration. Echocardiography demonstrated an atrial septal defect and a right subclavian artery arising from the aortic arch. The respiratory condition resolved, and the patient was discharged with plans for outpatient follow-up at the surgical clinic within 2–3 months to evaluate and schedule resection of the pulmonary sequestration.
